# A Hybrid Ant Colony Optimization and Dynamic Window Method for Real-Time Navigation of USVs

**DOI:** 10.3390/s25196181

**Published:** 2025-10-06

**Authors:** Yuquan Xue, Liming Wang, Bi He, Shuo Yang, Yonghui Zhao, Xing Xu, Jiaxin Hou, Longmei Li

**Affiliations:** 1 School of Electrical Engineering, Naval University of Engineering, Wuhan 430033, China; 21101501@nue.edu.cn (Y.X.); icesoar@163.com (L.W.); 1920191088@nue.edu.cn (Y.Z.); xuxin19901201@126.com (X.X.); 2020201040@nue.edu.cn (J.H.); vivianlee527@163.com (L.L.); 2 Shanghai Marine Equipment Research Institute, Shanghai 200030, China; wulin_xingic@163.com

**Keywords:** unmanned surface vehicle (USV), sensor-based navigation, ant colony optimization (ACO), dynamic window approach (DWA), real-time path planning, obstacle avoidance, multi-heuristic fusion

## Abstract

Unmanned surface vehicles (USVs) rely on multi-sensor perception, such as radar, LiDAR, GPS, and vision, to ensure safe and efficient navigation in complex maritime environments. Traditional ant colony optimization (ACO) for path planning, however, suffers from premature convergence, slow adaptation, and poor smoothness in cluttered waters, while the dynamic window approach (DWA) without global guidance can become trapped in local obstacle configurations. This paper presents a sensor-oriented hybrid method that couples an improved ACO for global route planning with an enhanced DWA for local, real-time obstacle avoidance. In the global stage, the ACO state–transition rule integrates path length, obstacle clearance, and trajectory smoothness heuristics, while a cosine-annealed schedule adaptively balances exploration and exploitation. Pheromone updating combines local and global mechanisms under bounded limits, with a stagnation detector to restore diversity. In the local stage, the DWA cost function is redesigned under USV kinematics to integrate velocity adaptability, trajectory smoothness, and goal-deviation, using obstacle data that would typically originate from onboard sensors. Simulation studies, where obstacle maps emulate sensor-detected environments, show that the proposed method achieves shorter paths, faster convergence, smoother trajectories, larger safety margins, and higher success rates against dynamic obstacles compared with standalone ACO or DWA. These results demonstrate the method’s potential for sensor-based, real-time USV navigation and collision avoidance in complex maritime scenarios.

## 1. Introduction

Unmanned surface vehicles (USVs), as autonomous platforms for ocean mapping, environmental monitoring, port inspection, and maritime security, rely heavily on multi-sensor systems—such as LiDAR, radar, GPS, inertial measurement units (IMUs), and cameras—to perceive their surroundings and ensure safe navigation [1,2]. These sensors provide critical information about static and dynamic obstacles, traffic conditions, and environmental uncertainty, enabling USVs to plan safe and efficient trajectories in real time [3,4,5]. However, converting raw sensor data into globally optimal and locally feasible paths remains a major challenge [6,7].

Path planning in sensor-driven USV navigation is typically organized into two layers [8]. Global path planning uses prior maps or sensor-fused environment models to produce optimal routes in relatively static settings [9], while local path planning depends on real-time perception for dynamic obstacle avoidance and trajectory refinement under sensor noise and environmental changes [10]. Relying solely on one layer often fails in cluttered waterways with narrow passages and moving obstacles. This has motivated research on integrating global and local planners to achieve both global efficiency and local reactivity for USVs in sensor-rich environments [11].

In recent years, researchers have explored a variety of advanced path planning techniques. For example, Bahwini et al. proposed a method for path planning in soft tissue deformation environments [12], which employs finite element modeling and bio-heat transfer analogy to explicitly account for environmental deformation and uncertainty, demonstrating strong adaptability in microscale scenarios. However, such approaches are primarily tailored for deformable media, which differ substantially from the large-scale rigid waterways encountered by unmanned surface vehicles (USVs), and they also incur considerable computational overhead. The optimal path planning method based on Cellular Neural Networks (CNNs) leverages parallel computation and dynamical system modeling, exhibiting strong nonlinear processing capability and distributed characteristics in complex environments [13]; nevertheless, the network scale and parameter sensitivity limit its applicability to large-scale maritime charts. In the field of unmanned aerial vehicles (UAVs), researchers have proposed the crossover recombination-based global-best Brainstorm Optimization algorithm (GBSO-CR), which enhances global exploration and diversity control to achieve superior paths in three-dimensional threat environments. Despite these advantages, its high computational complexity and strong parameter dependence restrict its suitability for real-time online path planning in USVs [14].

Overall, these approaches provide diverse strategies for addressing environmental uncertainty, dynamic changes, and real-time constraints. On the one hand, soft tissue deformation and thermal modeling methods are more applicable to small-scale or specific medium environments, making them difficult to adapt to maritime scenarios with rigid obstacles and large-scale continuous waterways. On the other hand, methods based on CNNs and brainstorm optimization, despite their strong global search and parallel processing capabilities, typically incur high computational costs and thus fall short of meeting the real-time planning requirements of USVs in dynamic marine environments.

Among global planners, Ant Colony Optimization (ACO) is popular for mobile robots and USVs thanks to its distributed search, positive feedback, and robustness in complex environments [15,16]. Yet the standard ACO exhibits several drawbacks in continuous spaces and densely cluttered scenes:

- Premature convergence: pheromones accumulate rapidly on a few paths, trapping the search in local optima.
- Insufficient safety: distance-only heuristics may drive paths that skim obstacle boundaries.
- Poor smoothness: without heading constraints, routes contain sharp turns that increase energy consumption and complicate control.
- Limited adaptability: success rates degrade in highly dynamic environments, and feasible paths may not be found.

For local planning, the Dynamic Window Approach (DWA) [17] evaluates motion commands directly in the robot’s (or USV’s) velocity space under kinematic constraints and is widely used for obstacle avoidance. However, classical DWA primarily relies on local sensing and lacks global guidance; it is prone to getting stuck in cul-de-sacs, concavities, or complex obstacle clusters, reducing efficiency and even preventing goal reachability [18]. Recent work has attempted to integrate improved ACO and DWA variants to combine global guidance with local reactivity. For example, Song et al. [19] proposed an ACO–DWA fusion strategy that enhances pheromone initialization and evaluation functions, achieving shorter and smoother paths for mobile robots in dynamic environments.

To address these issues, we propose a fusion method that couples an improved ACO with an enhanced DWA for USV path planning:

Global stage: the improved ACO fuses three heuristics—path length, obstacle clearance, and trajectory smoothness—into the transition rule, and adopts a cosine-annealed temperature to balance exploration and exploitation. A pheromone update with lower/upper bounds and a stagnation-triggered restart enhances convergence and diversity.

Local stage: the DWA cost is redesigned under USV kinematics by incorporating trajectory smoothness and goal-deviation terms, enabling dynamic tracking of the global reference path with real-time collision avoidance.

Fusion strategy: ACO supplies a safe and smooth global reference, while DWA performs trajectory optimization and avoidance around dynamics, preserving global optimality and local real-time feasibility.

The main contributions of our work are as follows:

(1) A multi-heuristic, temperature-controlled ACO that improves the safety and smoothness of global routes.
(2) A DWA cost tailored to USV motion characteristics, fusing speed, smoothness, and goal-deviation to realize dynamic avoidance with global-path tracking.
(3) An ACO–DWA integration that tightly couples global and local planning, markedly improving success rates in complex dynamic scenes.
(4) Comprehensive simulations showing that the proposed method outperforms standalone ACO and standalone DWA in path length, smoothness, safety margin, and avoidance success.

## 2. USV Kinematic Modeling

To incorporate a trajectory smoothness metric into the planner, we first establish a steering kinematic model for the USV [20]. Under a small-angle approximation and standard simplifications—neglecting sideslip and wave-induced disturbances—the planar motion of a USV can be treated as a nonholonomic system whose state is (*x*,*y*,*θ*), where *θ* is the heading.

**Figure 1:**
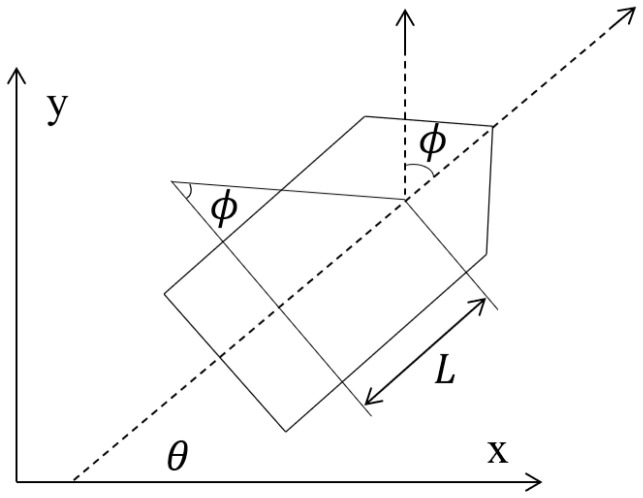
Simplified steering model.

As illustrated in Figure 1 (simplified steering model), assume the heading is aligned with the velocity direction. Let ϕ denote the rudder (steering) angle and L the distance from the center of mass to the steering center. The kinematics are then

(1) $$\left\{\begin{array}{l} \dot{x}=vcos\theta \\ \dot{y}=vsin\theta \\ \dot{\theta }=\frac{v}{L}tan\phi \end{array}\right.$$

where

v: longitudinal surge speed of the USV;

ϕ: rudder/steering angle;

L: distance from the center of mass to the steering center;

θ: heading angle.

## 3. Methods

### 3.1. Improved ACO

#### 3.1.1. Multi-Heuristic Fusion

In the standard ACO, the heuristic information $\eta_{ij}=1/d_{ij}$ only accounts for path length and thus fails to balance safety and smoothness. We introduce three factors—Distance, Clearance, and Smoothness—and fuse them with weights:

(2) $$\eta_{ij}^{\mathrm{fusion}}=w_{d}\cdot \frac{1}{d_{ij}+\varepsilon }+w_{c}\cdot \frac{clip\left(D_{j},0,D_{max}\right)}{D_{max}}+w_{s}\cdot \frac{1+cos\theta_{ij}}{2}$$

where

- $d_{ij}$: Euclidean distance from node i to node j;
- $D_{j}$: distance from node j to the nearest obstacle;
- $\theta_{ij}$: turning angle computed from the USV kinematic model;
- $w_{d},w_{c},w_{s}$: weights of the three factors;
- ε: a small constant to avoid division by zero;
- $clip\left(x,0,D_{\max}\right)=min\left\{max\{x,0\},D_{\max}\right\}$.

This fusion discourages “hugging” obstacles while favoring feasible paths with gentler heading changes.

#### 3.1.2. Temperature-Controlled Transition Probability

To balance exploration and exploitation adaptively, a temperature term T(t) modulates the influence of the heuristic:

(3) $$P_{ij}^{k}(t)=\frac{{\left[\tau_{ij}(t)\right]}^{\alpha }{\left[\eta_{ij}^{\mathrm{fusion}}\right]}^{\beta /T(t)}}{\sum_{l\in A_{k}}{\left[\tau_{il}(t)\right]}^{\alpha }{\left[\eta_{il}^{\mathrm{fusion}}\right]}^{\beta /T(t)}}$$

where $A_{k}$ is the selectable neighborhood for ant k. The temperature follows a cosine-annealing schedule:

(4) $$T(t)=T_{min}+\frac{T_{max}-T_{min}}{2}\left(1+cos\frac{\pi (t-1)}{t_{max}}\right)$$

At early iterations, T is larger, β/T is smaller, and the search is more exploratory; as T decreases, the algorithm shifts toward exploitation and stabilizes.

#### 3.1.3. Local Pheromone Update

When an ant traverses edge i→j, we apply a local “de-biasing” update:

(5) $$\tau_{ij}\leftarrow (1-\xi )\tau_{ij}+\xi \tau_{0},\;\xi \in (0,1)$$

which reduces over-commitment to recently used edges and increases diversity.

#### 3.1.4. Global Pheromone Update with Bounds

At the end of each iteration, pheromones evaporate and are reinforced by both ant contributions and an elite term:

(6) $$\tau_{ij}\leftarrow (1-\rho )\tau_{ij}+\Delta \tau_{ij}^{ant}+q(t)\Delta \tau_{ij}^{elite}$$

With

(7) $$\Delta \tau_{ij}^{\mathrm{ant}}=\sum_{k=1}^{m}\frac{Q}{L_{k}}1\left\{(i\to j)\in {\mathrm{path}}_{k}\right\},\;\Delta \tau_{ij}^{\mathrm{elite}}=\frac{Q}{L_{\mathrm{best}}}1\{(i\to j)\in \mathrm{best}\_\mathrm{path}\}$$

where $L_{k}$ is the length of ant k’s path and $L_{\mathrm{best}}$ is the current best length. We use a dynamic elite coefficient

(8) $$q(t)=exp\left(\frac{t-1}{t_{max}}\right)-1$$

and impose pheromone bounds

(9) $$\tau_{ij}\leftarrow min\left\{\tau_{max},max\left\{\tau_{min},\tau_{ij}\right\}\right\}$$

These mechanisms speed up convergence while preventing premature stagnation.

#### 3.1.5. Stagnation Criterion and Restart

If the best value shows no improvement for P consecutive iterations, we trigger a “pull-back” restart:

(10) $$\tau_{ij}\leftarrow (1-\gamma )\tau_{ij}+\gamma \tau_{0},\gamma \in (0,1)$$

which resets the pheromone field toward its initialization to restore exploration capability.

### 3.2. Pseudocode for the Improved ACO

At initialization (see Algorithm 1), pheromone levels $\tau_{ij}$ are set to $\tau_{0}$ and the fused heuristic $\eta_{ij}^{\mathrm{fusion}}$ in (2) is precomputed (or updated on demand) from the distance, clearance and smoothness terms. We fix $\alpha ,\beta ,\rho ,\xi ,\tau_{min},\tau_{max},\gamma$ and define the cosine-annealed temperature schedule T(t) in (4) together with the time-varying elite gain q(t) in (8). For each iteration $t=1,\ldots ,t_{max}$ (or until an early-stopping criterion is met), a colony of m ants independently constructs candidate paths using the transition rule (3) and applies the local update (5) along traversed edges. After all ants return, path costs are evaluated and the incumbent best solution is updated. Global pheromone evaporation and reinforcement are then performed using (6) and (7), followed by bounding in (9) to prevent premature saturation. If the best cost has not improved for P consecutive iterations, the stagnation pull-back in (10) is triggered to restore exploration. The loop records the periteration best cost for the convergence plot, and the final best path can be optionally post-processed by visibility-based node pruning or mild smoothing before being passed to the local planner. The environment map used for path construction can be either derived from sensor perception or generated by simulation.


**Algorithm 1**

**Improved-ACO (Global Planning)**
InputGrid map, start *s*, goal *g*, ACO parametersOutputbest_path, best_cost1Initialize pheromone $\tau \leftarrow \tau_{0}$ on all feasible edges.2Precompute distance field D and fused heuristic $\eta^{\mathrm{fusion}}$.3Set best_cost ←+∞, no_improve ←0.4for t=1 to $t_{max}$ do5

  perIterAntTau ←0; perIterElite ←0

6$\;\;\;T=T_{min}+\frac{T_{max}-T_{min}}{2}\left(1+cos\frac{\pi (t-1)}{t_{max}}\right)$.7   for k=1 to m do8$\;\;{path}_{k}\leftarrow \;\mathrm{ConstructPath}\_\mathrm{Pro}\;\left(M,s,g,\tau ,\eta^{\mathrm{fusion}},T,C_{\min},\xi ,\tau_{0},\alpha ,\beta \right)$.9

$\;\;{path}_{k}\leftarrow RemoveRedundant(\left.{path}_{k}\right))$

10

$\;\;L_{k}\leftarrow PathLength\left.\left({path}_{k}\right)\right)$

11

$\;\;\mathrm{perIterAntTau}\;\leftarrow \;\mathrm{perIterAntTau}+\Delta \tau ({path}_{k}\left.,L_{k},Q\right))$

12   end for13$\;\;\;\mathrm{if}\;\mathrm{all}\;L_{k}=\infty$ then14$\;\;\;C_{min}\leftarrow max\left(0,C_{min}-\Delta C\right)$; rerun this iteration15   if still infeasible then return failure16   end if17   Find best individual $\left(L^{*}\right.,\left.{path}^{*}\right)\;\mathrm{among}\left\{\left(L_{k}\right.\right.,\left.\left.{path}_{k}\right)\right\}$
18$\;\;\;\mathrm{if}\;L^{*}<$ best_cost then19

${\;\;\;best}_{cost}\leftarrow L^{*},best\_path\leftarrow {path}^{*},no\_improve\leftarrow 0$

20   else21   no_improve ← no_improve +122   end if23$\;\;\;perIterElite\leftarrow Q/L^{*}$ added only on edges of ${path}^{*}$
24

$\;\;\;q(t)\leftarrow exp\left(\frac{t-1}{t_{\max}}\right)-1$

25

   τ←(1−ρ)τ+perIterAntTau+q(t)·perIterElite

26

$\;\;\;\tau \leftarrow min\left\{\tau_{\max},max\left\{\tau_{\min},\tau \right\}\right\}$

27   if no_improve ≥P then28

$\;\;\;\tau \leftarrow (1-\gamma )\tau +\gamma \tau_{0}$

29

   no_improve ← 0

30   end if31end for32return best_path, best_cost

### 3.3. Simulation of the Improved ACO

We evaluate the proposed method in a 21 × 21 grid map with sparse obstacles, which emulate a simplified sensor-detected map. The maximum number of iterations is 250. After a path is generated, redundant waypoints are removed by a visibility-based triangle clipping procedure. The start and goal are set to (1,21) and (21,1), respectively. Baselines include several representative ACO variants: the original Ant System (AS) [21], Ant Colony Optimization (ACO) as a general framework [22], Ant Colony System (ACS) [23], Rank-based Ant System (RAS) [24], and Elitist Ant System (EAS) [21]. The proposed variant (“Ours”) is the improved ACO. Key parameter settings are summarized in Table 1.

**Figure 2:**
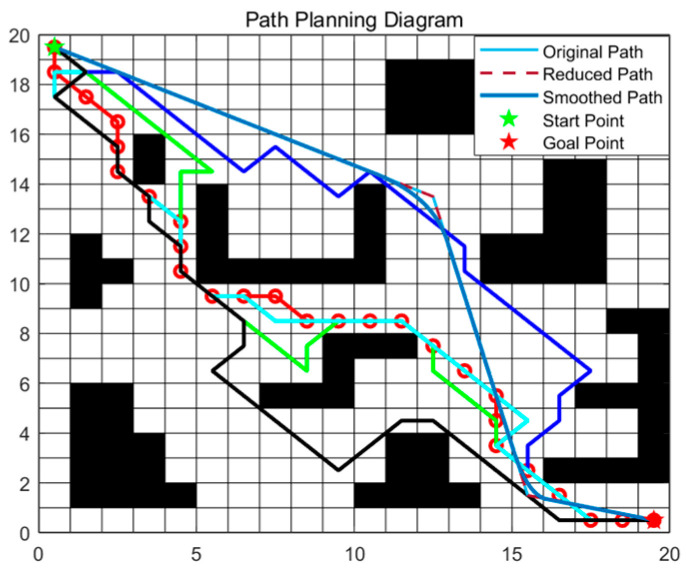
Compares the paths produced by different ACO variants.

**Figure 3:**
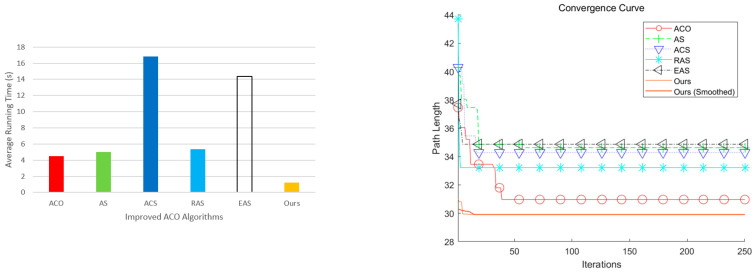
Runtime and convergence curves of different ACO variants.

In Figure 2, the red solid line with circles denotes vanilla ACO, the green curve AS, the dark-blue curve ACS, the cyan curve RAS, the black curve EAS, and the blue curve our improved ACO. The proposed method reduces fruitless branches during search and removes superfluous turns in the post-optimization stage, yielding markedly smoother trajectories. As summarized in Table 2, the average path length of the proposed method is 29.49—about 4.7% shorter than vanilla ACO—and its runtime is 1.24 s, which is lower than the baselines. Figure 3 further shows that our dynamic pheromone updating and node-pruning strategy leads to faster convergence.

### 3.4. Improved DWA

#### 3.4.1. Kinematic Model and Dynamic-Window Feasibility

Under a small-angle approximation and neglecting sideslip and wave disturbances, the USV’s planar motion follows the kinematics in (1), where the surge speed is aligned with the heading. Let v be the surge speed, ϕ the rudder (steering) angle, θ the heading, and L the distance from the vehicle’s CoG to the steering center. The minimum turning radius is given by the geometric relation

(11) $$R_{min}=\frac{L}{tan\varphi_{max}}$$

In practice, actuator saturation and hull dynamics impose bounds on speed, steering angle, and their rates. With sampling period Δt and current control ($v_{k},\phi_{k}$), the dynamic window is

(12) $$\begin{array}{l} v\in \left[max\left(v_{min},v_{k}+a_{min}\Delta t\right),min\left(v_{max},v_{k}+a_{max}\Delta t\right)\right]\\ \varphi \in \left[max\left(\varphi_{min},\varphi_{k}+{\dot{\varphi }}_{min}\Delta t\right),min\left(\varphi_{max},\varphi_{k}+{\dot{\varphi }}_{max}\Delta t\right)\right] \end{array}$$

To avoid numerical stagnation and deadlock, we enforce a low-speed threshold $v\geq v_{\epsilon }>0$. During sampling, candidates that violate curvature/steering feasibility (e.g., $|\phi |>\phi_{max}$ or equivalently κ=tanϕ/L beyond its bound) are discarded.

#### 3.4.2. Prediction Model and Safety Criterion

For each candidate control (v,ϕ), we roll out the kinematics (1) for $N_{p}$ steps to obtain the predicted trajectory

(13) $$\Gamma =\left\{\left(x_{l},y_{l},\theta_{l}\right)|l=1,2,\ldots ,N_{p}\right\}$$

Let the minimum Euclidean distance to all static/dynamic obstacles be

(14) $$d_{min}=\underset{l}{min}\;\underset{o\in O}{min}\;\left\|\left(x_{l},y_{l}\right)-\left(x_{o},y_{o}\right)\right\|$$

where O is the obstacle set and ($x_{o},y_{o}$) an obstacle position.

To couple hard safety with sensitivity near critical regions, we adopt a dual-threshold rule:

- Hard safety: if $d_{min}<D$ (hard safety radius), the candidate is infeasible and rejected.
- Soft safety: for $d_{min}\geq D$, a logistic cost enhances sensitivity near the soft boundary:
  (15) $${\mathrm{Clear}}^{*}=K\left(\frac{1}{1+exp\left(c\left(d_{min}-d_{lim}\right)\right)}-\frac{1}{2}\right)$$
  where $d_{lim}>D,c>0$ controls the slope, and K scales the penalty.

Around $d_{min}\approx d_{lim}$, the cost varies most rapidly, prompting early avoidance. Predicted positions of moving obstacles may be obtained via constant-velocity, constant-acceleration, or coordinated-turn models; our framework is agnostic to the predictor.

#### 3.4.3. Objective Function

To keep local avoidance consistent with the global objective, we maximize a multi-term score that blends goal heading, trajectory smoothness, clearance, speed preference, and relative goal bearing:

(16) $$\begin{array}{c} J(v,\phi )=\alpha Heading(v,\phi )+\beta Smooth(v,\phi )+\delta {Clear}^{*}(v,\phi )\\ +\gamma Velocity(v,\phi )+\mu RelAngle(v,\phi ) \end{array}$$

with nonnegative weights α,β,δ,γ,μ.

(1) Goal-heading term.

Let the local tracking target (e.g., the next point on the global path) be $\left(x_{g},y_{g}\right)$. With predicted terminal state ($x_{N_{p}},y_{N_{p}},\theta_{N_{p}}$), the desired bearing is

(17) $$\theta_{\mathrm{des}}=atan2\left(y_{g}-y_{N_{p}},x_{g}-x_{N_{p}}\right)$$

and the heading score is

(18) $$\mathrm{Heading}=\frac{1+cos\left(\theta_{N_{p}}-\theta_{des}\right)}{2}\in [0,1]$$

(2) Smoothness term.

To discourage sharp turns and improve maneuverability/efficiency,

(19) $$\mathrm{Smooth}=\frac{1}{N_{p}-1}\sum_{l=1}^{N_{p}-1}\;\frac{1+cos\left(\theta_{l+1}-\theta_{l}\right)}{2}\in [0,1]$$

(3) Clearance term.

If $d_{min}<D$ the candidate is rejected; otherwise the soft safety cost follows (16). For completeness we restate it:

(20) $${\mathrm{Clear}}^{*}=K\left(\frac{1}{1+exp\left\{c\left(d_{min}-d_{lim}\right)\right\}}-\frac{1}{2}\right)$$

(4) Speed preference.

Encouraging efficient motion via normalized surge speed,

(21) $$\mathrm{Velocity}=\frac{v-v_{min}}{v_{max}-v_{min}}\in [0,1]$$

(5) Relative goal-bearing term.

To re-align toward the goal after detours,

(22) $$\mathrm{RelAngle}=\frac{1+cos\left(\theta_{des}-\theta_{k}\right)}{2}\in [0,1]$$

where $\theta_{k}$ is the current heading.

### 3.5. Pseudocode of the Improved DWA

Outline. Initialize kinematic bounds and weights (α,β,δ,γ,μ). At each control cycle: (i) build the dynamic window (12) around ($v_{k},\phi_{k}$) and sample feasible (v,ϕ) respecting $|\phi |\leq \phi_{max}$ and $v\geq v_{\epsilon }$; (ii) roll out (1) for $N_{p}$ steps to get Γ; (iii) compute $d_{min}$ by (15); reject candidates with $d_{min}<D$, evaluate surviving ones by (16) with terms (19)–(22); (iv) select the maximizer of J and apply it; (v) advance the state and repeat. The detailed pseudocode is summarized in Algorithm 2.


**Algorithm 2**

**Improved-DWA (Local Planning with Hard/Soft Safety and Multi-Objective)**
InputInput: Current state x=(x,y,θ,v,ω), local goal, static map, predicted dynamic obstacles, DWA parameters.Output$\mathrm{Control}\;u^{*}=\left(v^{*},\phi^{*}\right),\;\mathrm{best}\;\mathrm{trajectory}\;trajj^{*}$.1$\mathrm{Build}\;\mathrm{dynamic}\;\mathrm{window}:\;v\in \left[max\left(v_{min},v_{k}+a_{min}\Delta t\right),min\left(v_{max},v_{k}+a_{max}\Delta t\right)\right];\phi \in \left[max\left(\phi_{min},\phi_{k}+{\dot{\phi }}_{min}\Delta t\right),min\left(\phi_{max},\phi_{k}+{\dot{\phi }}_{max}\Delta t\right)\right]$.2$\mathrm{Prune}\;\mathrm{samples}\;\mathrm{with}\;|\phi |>\phi_{max}\;\mathrm{or}\;v<v_{\varepsilon }$;3

$bestScore\leftarrow -\infty ;\;u^{*}\leftarrow \emptyset ;\;{traj}^{*}\leftarrow \emptyset ;$

4For each sample (v,ϕ) in dynamic window do5$\;\;\mathrm{Predict}\;N_{p}$-step trajectory $\Gamma ={\left\{\left(x_{l},y_{l},\theta_{l}\right)\right\}}_{l=1}^{N_{p}}$.6$\;\;d_{min}\leftarrow \underset{l}{min}\;\underset{o}{min}\;\left\|\left(x_{l},y_{l}\right)-\left(x_{o},y_{o}\right)\right\|$ over static + predicted obstacles.7$\mathrm{If}\;d_{min}<D$ then continue.8$\mathrm{Compute}\;\mathrm{desired}\;\mathrm{heading}\;\theta_{\mathrm{des}}=atan2\left(y_{g}-y_{N_{p}},x_{g}-x_{N_{p}}\right)$.9$\mathrm{Heading}=\frac{1+cos\left(\theta_{N_{p}}-\theta_{des}\right)}{2}$.10

$\mathrm{Smooth}=\frac{1}{N_{p}-1}\sum_{l-1}^{N_{p}-1}\;\frac{1+cos\left(\theta_{l+1}-\theta_{l}\right)}{2}$

11

${Clear}^{*}=K\left(\frac{1}{1+exp\left(c\left(d_{min}-d_{lim}\right)\right)}-\frac{1}{2}\right)$

12$\mathrm{Velocity}=\frac{v-v_{min}}{v_{max}-v_{min}}$.13$\mathrm{RelAngle}=\frac{1+cos\left(\theta_{\mathrm{des}}-\theta_{k}\right)}{2}$.14$J=\alpha \mathrm{Heading}+\beta \mathrm{Smooth}+\delta {Clear}^{*}+\gamma \mathrm{Velocity}+\mu \mathrm{RelAngle}$.15$\mathrm{If}\;J>\mathrm{bestScore}\;\mathrm{then}\;\mathrm{update}\;\mathrm{bestScore},\;u^{*}=(v,\phi ),traj^{*}=\Gamma$.16$\mathrm{Return}\;u^{*},traj^{*}$.

## 4. ACO-DWA Fusion

### 4.1. Algorithmic Flowchart

**Figure 4:**
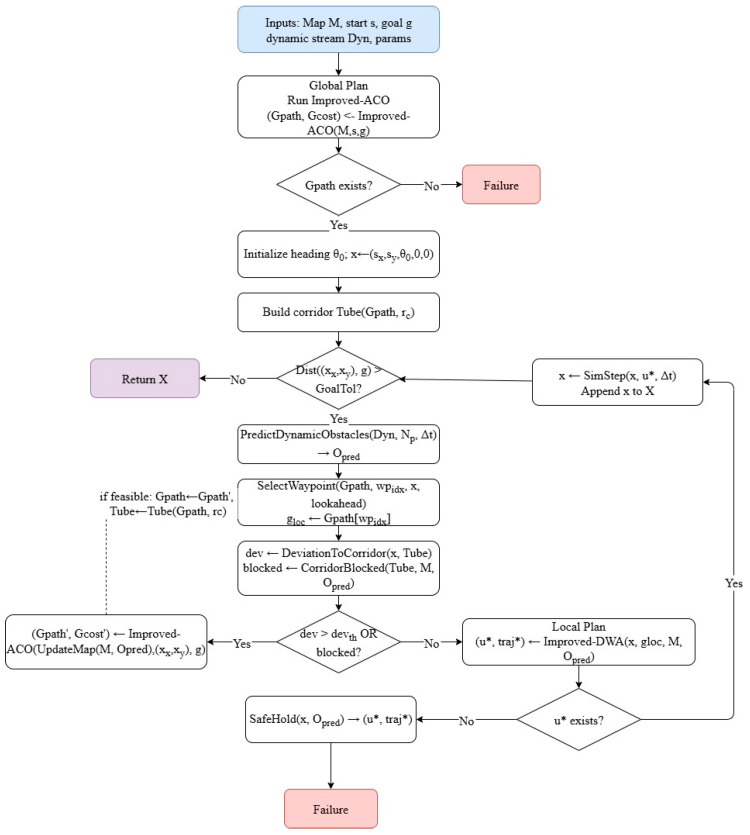
Flowchart of the proposed ACO–DWA hybrid path planner.

The overall procedure of the proposed global–local hybrid planner is summarized in a flowchart (Figure 4). In the global planning stage, the improved ACO constructs the grid graph and initializes both the pheromone distribution and fused heuristic function. Ant paths are iteratively generated through a temperature-controlled transition rule, while pheromone trails are updated locally and globally under bounded values, with a stagnation–restart mechanism to avoid premature convergence. Throughout this process, the best-so-far solution is retained as the candidate global path. To further enhance path quality, line-of-sight pruning and multi-segment polyline refinement are applied to remove redundant waypoints and reduce unnecessary heading changes, yielding a smooth global reference trajectory.

For local tracking, the improved DWA aligns the initial USV heading with the tangent of the first reference segment and constructs the dynamic window according to the vehicle’s kinematic constraints. Within this window, feasible velocity–yaw pairs (v,ϕ) are sampled and rolled out into short-horizon trajectories. Each candidate is evaluated using a composite cost function that balances goalheading alignment, smoothness, clearance, speed preference, and relative bearing. Unsafe trajectories are discarded, and the one with the highest score is executed. A receding update strategy is then adopted: the USV always tracks the sub-path starting from the closest reference waypoint and repeats the local control cycle until the goal is reached or a re-planning condition is triggered.

### 4.2. Simulation

**Figure 5:**
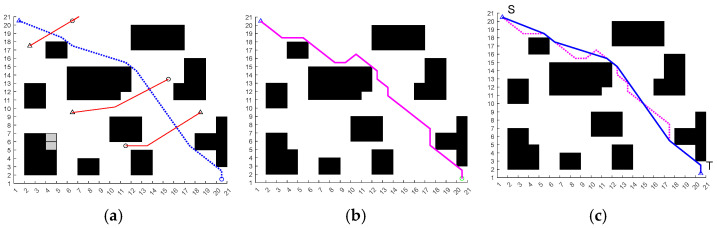
Global–local hybrid planning in a dynamic environment. (**a**) Three moving obstacles; (**b**) Initial planned path; (**c**) Optimized path.

This section validates the proposed global–local hybrid planner on a 20 × 20 grid environment (Figure 5). The map is generated from a predefined binary matrix, while the start and goal positions are interactively specified by the user. The scenario includes three moving obstacles, each following a polyline trajectory generated by the A* algorithm with a constant step size, as well as several additional static obstacles that are interactively placed to emulate unexpected obstacles detected by onboard sensors.

At the global layer, the improved ACO adopts the inverse squared Euclidean distance to the goal as the base heuristic, combined with time-varying evaporation and global pheromone reinforcement. The algorithm iterates to obtain a feasible discrete route, then applies line-of-sight pruning and multi-stage polyline refinement to reduce turns and produce a smooth global reference path. As illustrated in Figure 5, subfigure (a) shows the three moving obstacles, (b) depicts the initial planned path, and (c) presents the optimized global–local hybrid trajectory.

**Figure 6:**
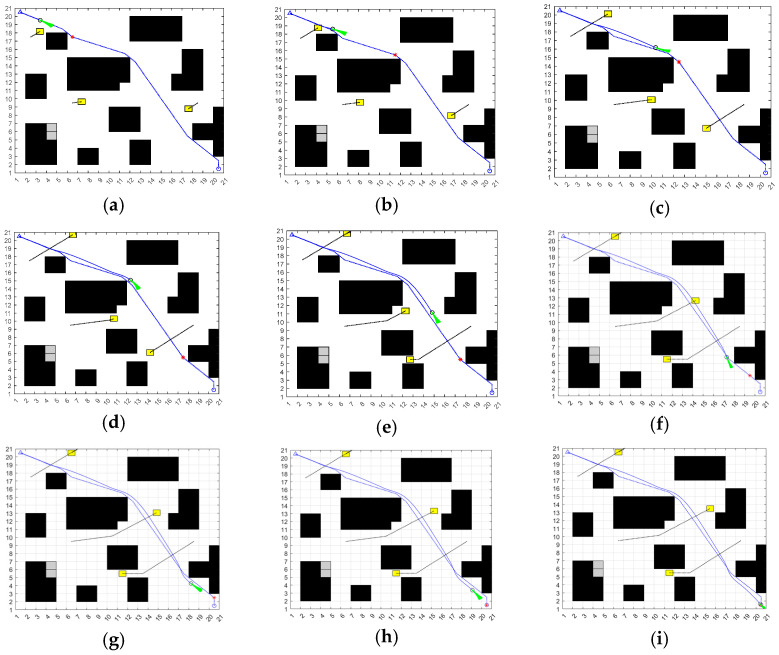
Sequential navigation process of the USV using the fused ACO–DWA planner. The blue dashed line represents the reference trajectory, and the blue solid line represents the actual trajectory. The triangle indicates the start position, and the green sector represents the forward heading direction. The red * marks the goal point. Hollow circles indicate intermediate waypoints. Black areas denote static obstacles, grey areas are expanded obstacles, and yellow blocks represent dynamic obstacles. (**a**) Start position and initial path planning; (**b**) Initial path tracking; (**c**) Passing the first obstacle cluster; (**d**) Mid-course turning and local adjustment; (**e**) Local adjustment near obstacles; (**f**) Exiting obstacle avoidance and continuing forward; (**g**) Approaching the goal region; (**h**) Entering the goal vicinity with fine adjustment; (**i**) Reaching the goal point.

At the local layer, the improved DWA automatically aligns the initial heading with the tangent of the first reference segment and tracks only the sub-path beginning at the nearest reference waypoint. Under dynamic/unknown obstacles, it samples feasible velocities in real time and evaluates a composite cost that blends heading, clearance, speed, and short-horizon prediction. Figure 6 presents the simulation results of the proposed ACO–DWA fusion algorithm. Subfigures (a)–(i) illustrate the sequential navigation states of the USV during its motion, where the trajectories remain feasible and collision-free under dynamic obstacles.

To further validate the effectiveness and robustness of the proposed planner, we conducted repeated trials under randomized obstacle configurations. Each method was executed 100 times, and performance was evaluated using four metrics: success rate, path length, minimum clearance, and goal time. The statistical results are summarized in Table 3. The proposed Improved ACO–DWA achieved the highest success rate (0.94), the shortest path length (28.3 m), the largest minimum clearance (0.57 m), and the shortest goal time (112.2 s). Compared with the baseline ACO + DWA, our method reduced path length by about 6% and increased minimum clearance by over 20%, while ensuring more efficient and safer navigation. These improvements are statistically significant (*p* < 0.05).

The improved ACO reaches a stable feasible solution within a small number of generations; after polyline refinement the path becomes significantly shorter and smoother. With the DWA fusion, the USV reaches the goal without collision, exhibiting smooth variations in speed and attitude throughout the motion.

## 5. Discussion

The integration of a multi-heuristic, temperature-controlled ACO with a kinematically feasible DWA produces trajectories that are short, smooth, and safe, while maintaining real-time responsiveness to dynamic obstacles. The combined heuristics of distance, clearance, and smoothness guide ACO away from wall-following shortcuts and abrupt heading changes; the cosine-annealed schedule stabilizes the exploration–exploitation balance, preventing early stagnation. Pheromone bounding, together with a stagnation–reset mechanism, further mitigates premature convergence.

On the local layer, aligning the initial heading with the first reference segment and tracking the nearest sub-path effectively reduce oscillations and dead ends. The redesigned DWA cost mirrors global objectives—heading consistency, clearance, smoothness, and velocity adaptability—thereby improving the continuity between global and local planning.

Computation remains modest through the use of dynamic ant counts, early stopping, array reuse, and parallel rollout. The DWA component operates at control frequency, enabling real-time deployment. The main limitations are the scalability of dense pheromone storage on large maps, and the absence of explicit modeling for currents and waves. The method also requires careful weight tuning. These limitations could be alleviated by sparse or learned roadmaps, disturbance-aware prediction, and automated parameter adaptation.

## 6. Conclusions

This paper presented a fused ACO–DWA planner for USV navigation that combines global optimality with local feasibility. The improved ACO generates safe and smooth global corridors, while the DWA executes collision-free tracking in the presence of dynamics and moving obstacles. Simulation experiments demonstrated faster convergence, shorter and smoother paths, and more stable real-time avoidance compared with classical ACO variants.

Future research will focus on improving scalability, modeling environmental disturbances such as currents and winds, automatic weight tuning, incorporation of formal safety guarantees (e.g., barrier functions or chance constraints), multi-USV cooperative planning, and validation on real-world sensor-based USV platforms.

## Figures and Tables

**Table 1:** Key parameters of the improved ACO.

Symbol	Meaning	Value
m	Number of ants	20
α	Pheromone importance	1.5
β	Heuristic importance	0.5
ρ	Evaporation rate	0.30
Q	Pheromone deposit constant	40
$t_{\max}$	Maximum iterations	250
$C_{\min}$	Minimum safety clearance (cells)	1.0

**Table 2:** Performance comparison across ACO variants.

Method	ACO	AS	ACS	RAS	EAS	Ours
Best iteration	10	1	6	2	2	3
Avg. path length	30.97	33.99	34.29	34.03	34.77	29.49
Runtime (s)	4.495	5.039	16.84	5.371	14.36	1.24
Smoothed	×	×	×	×	×	√

Note: √ indicates that path smoothing is applied; × indicates no smoothing.

**Table 3:** Statistical results of different methods (mean ± std; 95% CIs in parentheses).

Method	Success ↑	Path Length ↓ (m)	Min Clearance ↑ (m)	Goal Time ↓ (s)
ACO + DWA	0.88 (0.85–0.90)	30.1 ± 5.4 (28.4–31.8)	0.46 ± 0.10 (0.42–0.50)	118.2 ± 12.3 (113.7–122.7)
AS + DWA	0.90 (0.88–0.92)	29.6 ± 5.1 (28.0–31.2)	0.48 ± 0.10 (0.44–0.52)	116.0 ± 11.9 (111.7–120.3)
ACS + DWA	0.92 (0.90–0.94)	29.1 ± 5.0 (27.5–30.7)	0.50 ± 0.10 (0.46–0.54)	114.6 ± 11.5 (110.4–118.7)
RAS + DWA	0.92 (0.90–0.94)	28.9 ± 4.9 (27.3–30.5)	0.51 ± 0.10 (0.47–0.55)	114.1 ± 11.2 (110.0–118.2)
Ours	0.94 (0.92–0.95)	28.3 ± 4.8 (26.8–29.8)	0.57 ± 0.10 (0.53–0.61)	112.2 ± 10.8 (108.4–116.0)

Note: ↑ indicates that a larger value is better, whereas ↓ indicates that a smaller value is better.

## Data Availability

Data are contained within the article.
